# Association of shift work with metabolic dysfunction-associated fatty liver disease among subway workers

**DOI:** 10.3389/fpubh.2025.1737770

**Published:** 2025-12-29

**Authors:** Rong Peng, Bin Shi, Junling Liu, Zhenyu He

**Affiliations:** Department of Environmental Health, Wuhan Centers for Disease Prevention and Control, Wuhan, China

**Keywords:** body mass index, cross-sectional study, mediation effect, metabolic dysfunction-associated fatty liver disease, shift work

## Abstract

**Objective:**

Inconsistent associations between shift work and metabolic dysfunction-associated fatty liver disease (MAFLD) have been suggested. This study aimed to investigate the association between shift work characteristics and MAFLD in subway workers.

**Methods:**

This cross-sectional study was conducted in Wuhan, China, between December 2018 and January 2019, with 9,105 subway workers included after excluding participants with missing data on shift work or ultrasonography, with cancer, or with insufficient data to diagnose MAFLD. All participants were on-duty employees, covering various functional positions such as train drivers, station attendants, maintenance technicians, and administrative staff. Information on demographics, occupational history, and lifestyles was collected through standardized questionnaires. We used logistic regression models to estimate the association of shift work duration and types with MAFLD, and restricted cubic spline regression to examine the potential nonlinear relationship. Mediation analyses were employed to evaluate the potential mediating role of body mass index (BMI).

**Results:**

Compared with participants with no shift work, the multivariable-adjusted ORs (95% CIs) for those with ≤3, 3–6, and >6 years of shift work were 0.80 (0.68, 0.94), 1.21 (1.04, 1.41), and 1.60 (1.37, 1.88), respectively. A J-shaped relationship between shift work duration and MAFLD (*P*_overall_ < 0.001, *P*_nonlinear_ = 0.002) was observed, with the likelihood of MAFLD substantially increased after 3 years of shift work. Compared with participants with no shift work, MAFLD risk increased by 13% (OR:1.13, 95% CI: 0.95, 1.34), 22% (OR: 1.22, 95% CI: 1.05, 1.42), and 21% (OR: 1.21, 95% CI: 1.02, 1.42) for those worked in two-shift, three-shift, and four-shift, respectively. BMI adjustment attenuated these associations, with mediation analyses revealing significant mediation effects: mediation proportion was 48.5% (34.0, 64.0%) for shift work duration, 42.9% (5.0, 99.4%) for three-shift, and 47.5% (4.4, 111.5%) for four-shift systems (all *P* < 0.05).

**Conclusion:**

Both shift work duration and rotation systems were associated with MAFLD risk in subway workers, with BMI mediating approximately half of these relationships.

## 1 Introduction

Within the urban transportation systems, the expansion of subway networks has necessitated a growing workforce operating under continuous shift schedules to maintain uninterrupted service. However, emerging evidence suggests that such work patterns of rotating shiftwork may constitute a significant occupational health hazard, including cardiovascular diseases (1), metabolic disorders (2), and mental health issues (3). Among these health concerns, metabolic dysfunction-associated fatty liver disease (MAFLD) has become a major public health challenge (4), which is the most common cause of liver-related morbidity and mortality among adults worldwide (5), with a global prevalence estimated at 38% (6). Although shift work has been linked to MAFLD, the current epidemiological evidence remains inconclusive. Cross-sectional studies among Chinese (7) and Korean (8) steelworkers showed that >20 years of shift work was associated with increased risk of moderate–severe nonalcoholic fatty liver disease (NAFLD), while a prospective study based on the UK Biobank found that ≥10 years of night shift work was associated with a 51% higher NAFLD risk (9). However, contrasting findings from the National Health and Nutrition Examination Survey showed no significant association (10), highlighting the need for further research.

Crucially, existing studies have largely overlooked the structural variability inherent in shift work systems, such as two-shift, three-shift, and four-shift rotations, each imposing distinct circadian and metabolic strains. Moreover, subway workers represent a uniquely exposed yet understudied occupational group, characterized by high-frequency night shifts, prolonged tenure in rotating schedules, and operational demands that differentiate them from other shift-working populations. No previous study has comprehensively examined the dose–response relationship between shift duration and MAFLD risk in this population, nor systematically compared the impacts of different shift systems.

To address these evidence gaps, this study investigates the association between rotating shift work, considering both duration and system type, and MAFLD among subway workers. We further evaluate the potential mediating role of body mass index. By focusing on a large, homogeneous occupational group with well-characterized and heterogeneous shift exposures, this research aims to provide novel insights into schedule-related metabolic risk and inform targeted workplace health strategies.

## 2 Methods

### 2.1 Study population

A total of 11,960 subway workers from Wuhan Metro Group Co., Ltd. were recruited and completed questionnaires between December 2018 and January 2019. In the present study, we excluded participants who had missing data on shift work (*n* = 518), had cancer (*n* = 8), without liver ultrasound examination (*n* = 2,321), or with insufficient data to diagnose MAFLD (*n* = 17). Thus, a total of 9,105 participants were included in the analyses. Detailed information on participant selection is depicted in Supplementary Figure S1.

All participants provided written informed consent, and this study was approved by the Wuhan Center for Disease Prevention and Control Ethics Committee (WHCDCIRB-K-2018042).

### 2.2 Ascertainment of shift work

Shift work was defined as having any work schedule involving irregular working hours instead of a normal daytime work schedule (11). Participants were asked, “Does your work involve shift work?” (yes/no). The participants who answered “yes” were then asked to specify their shift work type (two-shift, three-shift, or four-shift) and the number of years they had been engaged in shift work. In the two-shift system, employees work either a day shift (08:00–17:00) or a night shift (20:00–05:00), 5 days a week, with a one-month shift cycle. In the three-shift system, employees work one of the three shifts: morning shift (06:00–14:00), evening shift (14:00–22:00), or night shift (22:00–06:00). Workers rotate through these shifts over a 3-week cycle, working 5 days a week. For example, an employee might work the morning shift in the first week, the evening shift in the second week, the night shift in the third week, and then repeat the cycle, starting with the morning shift again. In the four-shift system, employees work rotate through a morning shift (06:00–14:00), an evening shift (14:00–22:00), a night shift (22:00–06:00), and a 24-h rest shift. The shift cycle is 4 days, with each shift lasting one day followed by 3 days off. Temporary substitutions may occur in cases of staff shortages. Self-reported shift work duration was categorized as ≤3 years, 3–6 years, and >6 years.

### 2.3 Assessment of covariates

Covariates, including age, sex, educational status (high school or below, university/college, or graduate student or above), smoking and drinking status (current, past, or never), dietary habits, sleep duration, sleep quality, and active exercise, were obtained by questionnaire. Participants who smoked at least one cigarette per day for the past 6 months were defined as current smokers. Participants who drank ≥1 time/week over the past half a year were defined as current drinkers. Active exercise was defined as engaging in moderate or moderate-vigorous intensity activities ≥150 min/week or engaging in vigorous-intensity activities ≥75 min/week (12). Body mass index was calculated by weight (kg) divided by height (m) squared. Hypertension was identified through a combination of self-reported medical history, ongoing treatment with antihypertensive drugs, or blood pressure ≥140/90 mm Hg (13). Dyslipidemia was diagnosed based on self-reported medical history, active lipid-lowering therapy, or specific lipid profile thresholds: total cholesterol ≥6.22 mmol/L, high-density lipoprotein cholesterol <1.04 mmol/L, low-density lipoprotein cholesterol ≥4.14 mmol/L, or triglycerides ≥2.26 mmol/L (14). Diabetes was ascertained by self-reported medical history, the presence of anti-diabetic medication, or fasting blood glucose ≥7.0 mmol/L (15). We grouped 26 types of jobs into four categories: technicians (including vehicle maintenance workers, telecommunications workers, signal workers, etc.), train drivers, service workers (including station attendants, ticketing administrators, etc.), and office workers (including managers, clerical support workers, etc.).

### 2.4 Ascertainment of MAFLD

The abdominal ultrasound examination is conducted by experienced sonographers using high-resolution ultrasound machines. MAFLD was defined as abdominal ultrasonography-diagnosed fatty liver disease along with the presence of one of the following three criteria: overweight/obesity (BMI ≥ 23 kg/m^2^ in Asians), presence of diabetes mellitus, or evidence of metabolic dysregulation (16). The metabolic dysregulation was defined as the presence of at least 2 of the following metabolic risk abnormalities: ① blood pressure ≥130/85 mmHg or receiving antihypertensives, ② plasma triglyceride ≥1.70 mmol/L or receiving specific drug treatment, ③ plasma high-density lipoprotein cholesterol <1.0 mmol/L for men and <1.3 mmol/L for women or receiving specific drug treatment, ④ prediabetes defined as having fasting glucose 5.6 to 6.9 mmol/L (16).

### 2.5 Statistical analysis

Continuous variables were presented as mean ± SD, and categorical variables were presented as frequency (percentage). Differences between groups were compared using student *t-*test for continuous variables and chi-square test for categorical variables.

Logistic regression models were used to assess the association of shift work with MAFLD. Model 1 adjusted for age, sex, educational status, smoking status, drinking status, sleep duration, sleep quality, dietary consumption of grain, beans or soy products, vegetables and fruits, milk or dairy products, meat, fish or seafood, and egg, and job category; model 2 further adjusted for hypertension, dyslipidemia, and diabetes. To explore the association between shift work and MAFLD independent of BMI, we additionally adjusted for BMI based on model 2. Missing data of continuous variables were imputed with the median value, and a dichotomous variable was created to flag the missingness, and an extra category was added to denote the missingness for categorical variables. Restricted cubic spline regression was employed to evaluate the potential nonlinear relationship between the shift work duration and MAFLD. As a previous study indicated that BMI may mediate the association between shift work and MAFLD (9), our study revealed that the association was substantially attenuated after additionally adjusting for BMI, which provided preliminary support for the plausible mediating role of BMI. Thus, we used the R package “mediation” (17) to estimate the potential mediation effect of BMI between shift work and MAFLD. The mediated proportion reflected the average mediation effect of BMI. To explore potential heterogeneity, stratified analyses were performed by sex (male or female), BMI [<24 or ≥24 kg/m^2^ according to the standard classification specific for the Chinese population (18)], current smokers (yes or no), current drinkers (yes or no), sleep duration (≤7 or >7 h), hypertension (yes or no), dyslipidemia (yes or no), and job category (technicians, train drivers, service workers, or office workers). The interactions were tested by adding multiplicative interaction terms into the models. Sensitivity analysis was conducted by excluding participants with missing values.

Analyses were conducted using R software (version 4.2.2, R Core Team) or SAS program (version 9.4, SAS Institute).

## 3 Results

### 3.1 Characteristics of the study population

Among the 9,105 participants eligible for the analyses, 2,590 had MAFLD. The mean age of the participants was 27.2 ± 4.2 years, and 20.0% were women. Subjects with MAFLD were older, more likely to be male, had higher prevalences of current smoking and drinking, with higher BMI and shorter sleep duration, and were more likely to have hypertension, dyslipidemia, and diabetes (Table 1). 70.4% of the participants were involved in shift work. Participants who worked in shifts were younger, more likely to be female, less likely to be smokers and drinkers, exercised inactively, had longer sleep duration, and were less likely to have dyslipidemia and diabetes (Supplementary Table S1).

**Table 1:** Basic characteristics of the study population.

Variables	Overall	Non-MAFLD	MAFLD	*P-*value^a^
*N*	9,105	6,515	2,590	
Age, years	27.2 ± 4.2	26.7 ± 4.0	28.5 ± 4.6	<0.001
Female, *n* (%)	1820 (20.0)	1,677 (25.7)	143 (5.5)	<0.001
Body mass index^b^, kg/m^2^	23.3 ± 3.7	21.8 ± 2.6	27.2 ± 3.0	<0.001
Education level^b^, *n* (%)				0.14
High school or below	47 (0.5)	36 (0.6)	11 (0.4)	
University/college	8,773 (96.4)	6,257 (96.0)	2,516 (97.1)	
Graduate student or above	182 (2.0)	141 (2.2)	41 (1.6)	
Smoking status^b^, *n* (%)				<0.001
Never smoker	6,824 (74.9)	5,130 (78.7)	1,694 (65.4)	
Current smoker	1972 (21.7)	1,177 (18.1)	795 (30.7)	
Former smoker	284 (3.1)	188 (2.9)	96 (3.7)	
Drinking status^b^, *n* (%)				<0.001
Never drinker	7,244 (79.6)	5,287 (81.2)	1957 (75.6)	
Current drinker	1,658 (18.2)	1,098 (16.9)	560 (21.6)	
Former drinker	182 (2.0)	114 (1.7)	68 (2.6)	
Active exercise^b^, *n* (%)	1,295 (14.2)	916 (14.1)	379 (14.6)	0.58
Sleep duration^b^, h	7.6 ± 1.5	7.7 ± 1.5	7.3 ± 1.4	<0.001
Sleep quality^b^, *n* (%)				0.27
Good	2,174 (23.9)	1,567 (24.1)	607 (23.4)	
Fair	5,057 (55.5)	3,653 (56.1)	1,404 (54.2)	
Poor	1,077 (11.8)	752 (11.5)	325 (12.5)	
Diet categories (≥5 times/week)^b^, *n* (%)				
Grain	8,881 (97.5)	6,358 (97.6)	2,523 (97.4)	0.98
Beans or soy products	2,402 (26.4)	1744 (26.8)	658 (25.4)	0.23
Vegetables and fruits	7,798 (85.6)	5,597 (85.9)	2,201 (85.0)	0.69
Milk or dairy products	3,242 (35.6)	2,352 (36.1)	890 (34.4)	0.17
Meat	7,513 (82.5)	5,331 (81.8)	2,182 (84.2)	0.002
Fish or seafood	1,589 (17.5)	1,145 (17.6)	444 (17.1)	0.74
Egg	4,048 (44.5)	2,883 (44.3)	1,165 (45.0)	0.45
Shift work duration, *n* (%)				<0.001
No shift work	2,699 (29.6)	1852 (28.4)	847 (32.7)	
≤3 years	2,481 (27.2)	2031 (31.2)	450 (17.4)	
3–6 years	2,407 (26.4)	1746 (26.8)	661 (25.5)	
>6 years	1,518 (16.7)	886 (13.6)	632 (24.4)	
Shift work type, *n* (%)				<0.001
No shift work	2,699 (29.6)	1852 (28.4)	847 (32.7)	
Two-shift	1,095 (12.0)	728 (11.2)	367 (14.2)	
Three-shift	3,685 (40.5)	2,747 (42.2)	938 (36.2)	
Four-shift	1,626 (17.9)	1,188 (18.2)	438 (16.9)	
Job category, *n* (%)				<0.001
Maintenance work	4,307 (47.3)	2,886 (44.3)	1,421 (54.9)	
Service work	1,299 (14.3)	1,094 (16.8)	205 (7.9)	
Driver	1,345 (14.8)	935 (14.4)	410 (15.8)	
Office work	2,154 (23.7)	1,600 (24.6)	554 (21.4)	
Hypertension^b^, *n* (%)	893 (9.8)	413 (6.3)	480 (18.5)	<0.001
Dyslipidemia^b^, *n* (%)	1,662 (18.3)	586 (9.0)	1,076 (41.5)	<0.001
Diabetes^b^, *n* (%)	91 (1.0)	25 (0.4)	66 (2.5)	<0.001

MAFLD, metabolic dysfunction-associated fatty liver disease. Continuous variables are presented as mean ± SD. Categorical variables are presented as numbers (percentages). ^a^*P-*values were estimated using student t test for continuous variables, and Chi-square tests for categorical variables. ^b^Data were incomplete for these variables. A total of 21 (0.2%), 103 (1.1%), 25 (0.3%), 21 (0.2%), 201 (2.2%), 141 (1.6%), 797 (8.8%), 125 (1.4%), 553 (6.1%), 336 (3.7%), 493 (5.4%), 373 (4.1%), 565 (6.2%), 474 (5.2%), 544 (6.0%), 501 (5.5%), and 608 (6.7%) participants had missing value of BMI, education level, smoking status, drinking status, active exercise, sleep duration, sleep quality, dietary consumption of grain, beans or soy products, vegetables and fruits, milk or dairy products, meat, fish or sea food, and egg, hypertension, dyslipidemia, and diabetes.

### 3.2 Association of shift work duration and type with MAFLD

**Figure 1:**
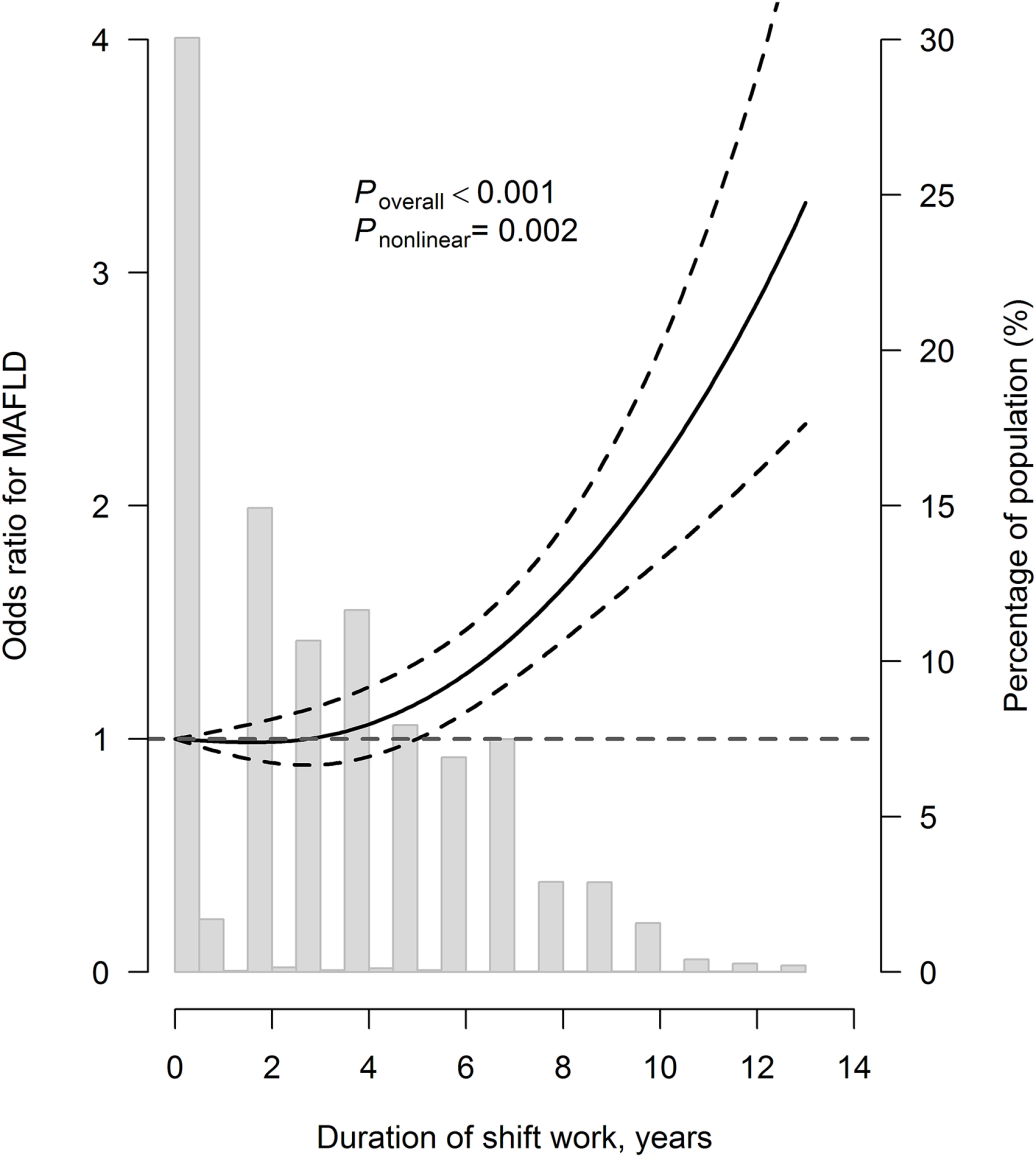
The restricted cubic spline curve for the association of shift work duration with metabolic dysfunction-associated fatty liver disease. Model adjusted for age, sex, educational status, smoking status, drinking status, sleep duration, sleep quality, dietary consumption of grain, beans or soy products, vegetables and fruits, milk or dairy products, meat, fish or seafood, and egg, job category, hypertension, dyslipidemia, and diabetes. The solid line represents adjusted odds ratios according to the change of shift work duration, while the dotted lines represent the 95% confidence intervals. Knots were placed at the 5th, 50th, and 95th percentiles, with the 10th percentile set as reference.

As shown in Table 2, compared with individuals with no shift work, the risk of MAFLD decreased by 18% (OR: 0.82, 95% CI: 0.70, 0.95) for those with ≤3 years of shift work, and increased by 18% (OR: 1.18, 95% CI: 1.03, 1.36) and 49% (OR: 1.49, 95% CI: 1.29, 1.73) for those with >3–6 and >6 years of shift work, respectively, after adjustments for demographics and lifestyles. When additionally adjusting for hypertension, dyslipidemia, and diabetes, the ORs (95% CIs) were 0.80 (0.68, 0.94), 1.21 (1.04, 1.41), and 1.60 (1.37, 1.88), respectively. For every 1-year increase in shift work, the risk of MAFLD increased by 6% (OR: 1.06, 95% CI: 1.04, 1.08). When further adjusted for BMI, the association was slightly attenuated. Restricted cubic spline regression analysis showed a J-shaped relationship between the shift work duration and MAFLD (*P*_overall_ < 0.001, *P*_nonlinear_ = 0.002; Figure 1), with the likelihood of MAFLD substantially increased after 3 years of shift work.

**Table 2:** Association of shift work with metabolic dysfunction-associated fatty liver disease.

Variables	Cases/total population	Model 1		Model 2		Model 2 + BMI	
		OR (95% CI)	*P* value	OR (95% CI)	*P-*value	OR (95% CI)	*P-*value
Shift work duration, years							
No shift work	847/2699	1.00 (Ref)		1.00 (Ref)		1.00 (Ref)	
≤3	450/2481	0.82 (0.70, 0.95)	0.01	0.80 (0.68, 0.94)	0.008	0.82 (0.66, 1.03)	0.09
>3–6	661/2407	1.18 (1.03, 1.36)	0.02	1.21 (1.04, 1.41)	0.01	1.23 (1.01, 1.49)	0.04
>6	632/1518	1.49 (1.29, 1.73)	<0.001	1.60 (1.37, 1.88)	<0.001	1.49 (1.21, 1.84)	<0.001
per 1 year increase	2590/9105	1.05 (1.04, 1.07)	<0.001	1.06 (1.04, 1.08)	<0.001	1.06 (1.03, 1.08)	<0.001
Shift work type							
No shift work	847/2699	1.00 (Ref)		1.00 (Ref)		1.00 (Ref)	
Two-shift	367/1095	1.14 (0.98, 1.34)	0.10	1.13 (0.95, 1.34)	0.16	1.08 (0.86, 1.36)	0.50
Three-shift	938/3685	1.17 (1.02, 1.35)	0.03	1.22 (1.05, 1.42)	0.01	1.24 (1.01, 1.51)	0.04
Four-shift	438/1626	1.15 (0.99, 1.34)	0.07	1.21 (1.02, 1.42)	0.02	1.22 (0.98, 1.51)	0.08

Model 1 adjusted for age, sex, education level, smoking status, drinking status, active exercise, sleep duration, sleep quality, dietary consumption of grain, beans or soy products, vegetables and fruits, milk or dairy products, meat, fish or seafood, and egg, and job category. Model 2 additionally adjusted for hypertension, dyslipidemia, and diabetes.

Compared with participants with no shift work, individuals who worked two-shift, three-shift, and four-shift were associated with 13% (OR: 1.13 95% CI: 0.95, 1.34), 22% (OR: 1.22, 95% CI: 1.05, 1.42), and 21% (OR: 1.21, 95% CI: 1.02, 1.42) higher risk of MAFLD, respectively, after full adjustments for demographics, lifestyles, and preexisting chronic conditions. When further adjusted for BMI, the association was attenuated. In the sensitivity analyses, excluding participants with missing values did not materially change the results (Supplementary Table S2).

### 3.3 Mediation effect of BMI on the association between shift work and MAFLD

**Figure 2:**
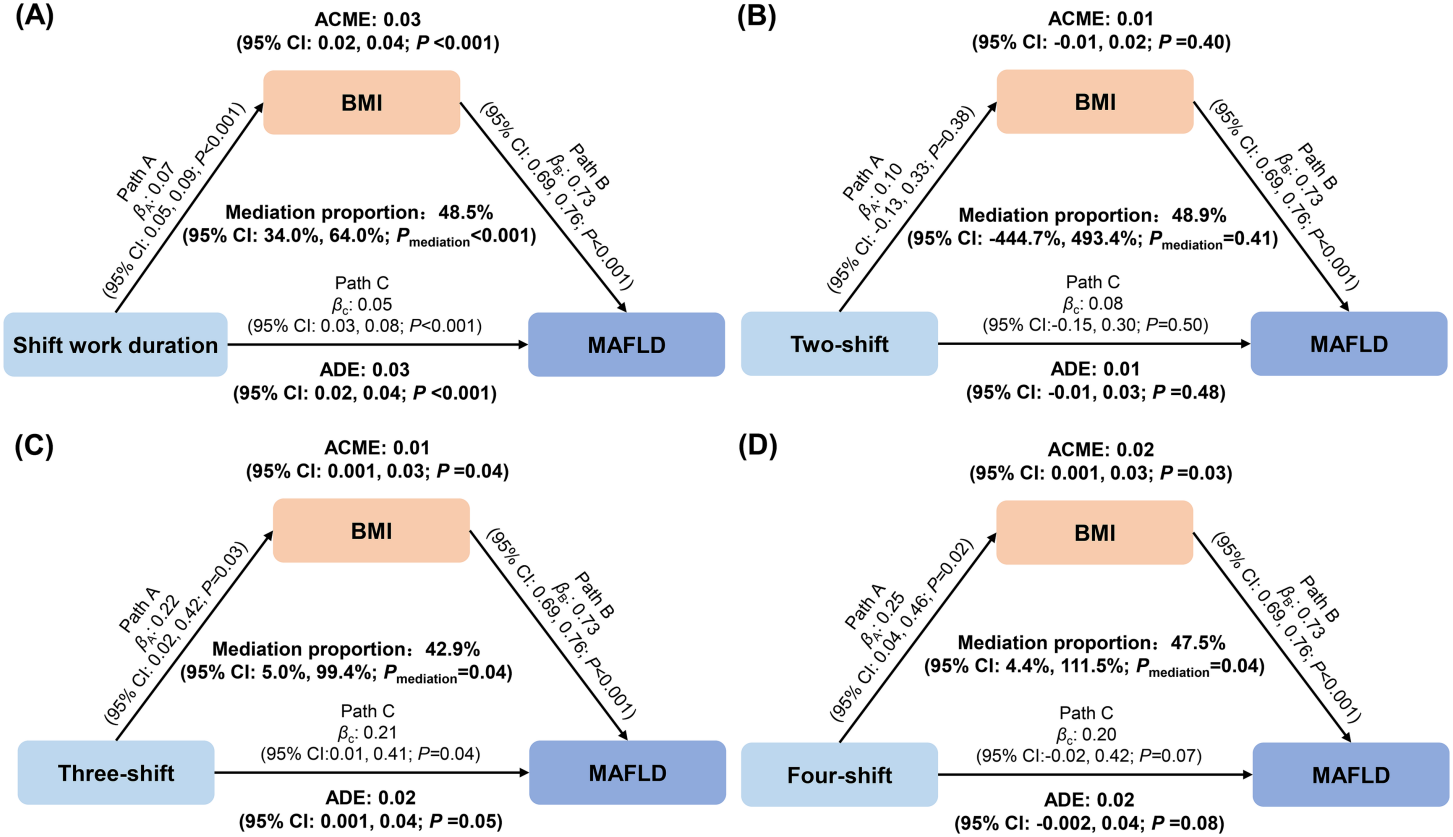
Mediation effects of body mass index on the associations of shift work duration and types with metabolic dysfunction-associated fatty liver disease.**(A)** shift work duration; **(B)** two-shift; **(C)** three-shift; **(D)** four-shift.The mediation analyses were performed using the R package “mediation” with 1,000 simulations. Mediation models were adjusted for age, sex, educational status, smoking status, drinking status, sleep duration, sleep quality, dietary consumption of grain, beans or soy products, vegetables and fruits, milk or dairy products, meat, fish or seafood, and egg, job category, hypertension, dyslipidemia, and diabetes. Outcome models were additionally adjusted for body mass index. ACME, average causal mediation effects; ADE, average direct effects.

As an established risk factor for MAFLD, we observed that BMI was significantly associated with shift work duration (*β*: 0.07, 95% CI: 0.05, 0.09), three-shift (β: 0.22, 95% CI: 0.02, 0.42), and four-shift (β: 0.25, 95% CI: 0.04, 0.46) systems. As shown in Figure 2, we found that BMI mediated a separate 48.5% (34.0, 64.0%), 42.9% (5.0, 99.4%), and 47.5% (4.4, 111.5%) of the association between shift work duration, three-shift system, four-shift system, and MAFLD, respectively.

### 3.4 Stratified analyses

In the analyses stratified by job category, significant heterogeneity was observed in the association between shift work duration and the risk of MAFLD prevalence (*P*_interaction_ = 0.02). For every 1-year increase in shift work, ORs (95% CIs) for maintenance workers, service workers, drivers, and office workers were 1.03 (1.00, 1.05), 1.15 (1.05, 1.25), 1.19 (1.09, 1.29), and 1.07 (1.04, 1.11), respectively (Supplementary Figure S2). Stratified analyses also suggested that the association between shift work duration and MAFLD was more pronounced among participants who slept≥7 h per night (*P*_interaction_ = 0.03). Other stratified analyses yielded no significant interactions (Supplementary Tables S3, S4).

## 4 Discussion

To our knowledge, this is the first study that comprehensively explores the association of shift work with MAFLD among subway workers, a group with chronic and intensive occupational exposure to shift work, but has been less studied. In this study, more than 70% of the participants were involved in shift work. We found that the shift work duration was associated with MAFLD in a J-shaped manner, and participants with >3 years of shift work had a higher risk of MAFLD. In addition, participants who worked in a three-shift system or a four-shift system experienced an elevated risk of MAFLD. Moreover, BMI mediated approximately 40–50% of the observed associations. These results extend current understanding of occupational health risks in critical infrastructure workers.

Limited prior research has examined the association between shift work duration and NAFLD, and our findings align with and extend existing evidence. A cross-sectional study conducted among 2,511 Korean steelworkers indicated that individuals worked in shifts for ≥20 years had a 2.86 times higher risk of moderate–severe NAFLD than those working daytime (8), and another study based on 6,881 Chinese steelworkers found that individuals had a 38% higher risk of moderate–severe NAFLD at similar shift work durations (7). A prospective study performed using data from the UK Biobank found that ≥10 years of night shift work was associated with a 51% higher risk of NAFLD (9). Notably, our study identified risk elevation at substantially shorter durations (>3 years). This accelerated risk onset may reflect the synergistic effects of circadian disruption due to persistent night shifts, high psychological and physical workloads, and distinctive confined underground environmental conditions of subway operations. The protective association observed during initial exposure periods (≤3 years) likely represents the “healthy worker effect” (19), as the company screened the physical conditions and ages of the workers before employment, and the physical fitness of the workers engaging in shift work was better than that of the general population, resulting in lower morbidity of diseases in the first few years of work.

In the present study, we also found that workers engaging in three-shift or four-shift systems were associated with a higher risk of MAFLD, which aligns with our previous study showing that the three-shift and four-shift systems were associated with elevated alkaline phosphatase levels (20). As alkaline phosphatase serves as a sensitive biomarker for hepatobiliary dysfunction, its elevation may suggest potential disturbances in liver metabolism and biliary function resulting from rotational shift work. However, studies evaluating the association between different shift work systems and MAFLD remain limited. Most existing literature tends to treat “shift work” as a homogeneous exposure, overlooking critical variations among shift patterns in terms of night shift frequency, rotation speed, and recovery intervals, all factors that may differentially impact circadian stability and metabolic health (21). Thus, our detailed analysis of shift system heterogeneity provides new insights into how specific work arrangements affect liver health. Future studies with a large sample size and long follow-up duration are warranted to verify the relationship between different shift patterns and MAFLD.

The mechanisms underlying the association between shift work and MAFLD remain largely unknown. In line with the previous study that showed BMI mediated 28.9% of the association between night shift work and NAFLD (9), our study also observed such a mediation role of BMI, indicating that adiposity may serve as an important pathway through which night shift work contributes to MAFLD. Meanwhile, the underlying mechanisms likely involve multiple interconnected physiological disruptions, with circadian rhythm disturbance serving as a central pathway. Night-oriented shift systems, particularly three-shift and four-shift arrangements characterized by frequent night work and inadequate recovery periods, can induce profound circadian misalignment (21). This misalignment affects fundamental metabolic processes through altered meal timing, dysregulated clock gene expression, and disturbed hormone secretion, collectively impairing metabolic homeostasis and promoting weight gain and hepatic fat accumulation (22). Beyond circadian disruption, other conditions, including poor dietary patterns marked by excessive energy intake and reduced nutritional quality (23, 24), activation of the hypothalamic–pituitary–adrenal axis, insulin resistance (25, 26), inflammation (27), and oxidative stress (28) may also serve as possible pathways. Given the widespread prevalence of shift work and its potential impact on metabolic health, further investigation into the mechanisms underlying the association between shift work and MAFLD is warranted to inform targeted interventions and improve the health of shift workers.

This study has some strengths, including a large sample, standardized ultrasonographic MAFLD diagnosis, and comprehensive evaluation of both shift work duration and systems. However, our study also has several limitations. First, the cross-sectional design of our study inherently limits causal inference regarding the associations among shift work, BMI, and MAFLD. Individuals with metabolic dysfunction or MAFLD may have transferred out of demanding shift schedules, which may introduce reverse causation and attenuate the observed associations in our study. In addition, although a mediating role of BMI in the association between shift work and MAFLD was observed, which suggests a potential mechanistic link, it is important to recognize that BMI itself is not a definitive biological mechanism but rather a composite indicator reflecting complex interactions of lifestyle factors (e.g., dietary patterns, physical activity) and metabolic alterations. Therefore, our findings should be interpreted as a statistical mediation rather than a demonstration of causal mechanism. Future research employing longitudinal designs or Mendelian randomization approaches would be valuable in clarifying the temporal sequence and establishing a causal relationship. Second, information on shift work and lifestyles (e.g., smoking, alcohol consumption, sleep duration, and dietary habits) was collected via self-report, while metabolic conditions (hypertension, dyslipidemia, and diabetes) were ascertained through a combination of laboratory measurements, self-reported physician diagnoses, and medication records, the potential for misclassification remains across all variables. Meanwhile, although we adjusted for dietary consumption of main food categories, the amount of energy intake and dietary pattern, which have a major impact on MAFLD, were not available in our study and could not completely rule out the possible effect of dietary factors on MAFLD. Moreover, although we have accounted for several known confounding factors, we cannot fully exclude residual confounding.

In conclusion, we found that participants with >3 years of shift work, and those worked in three-shift and four-shift were associated with a higher risk of MAFLD, with BMI mediating approximately half of the observed relationships. Our findings underscore the need for evidence-based shift work scheduling policies and targeted weight management programs for the prevention of MAFLD among shift workers in 24/7 transportation systems. Future studies are warranted to validate the association between shift work and MAFLD and evaluate the potential mechanisms of these findings.

## Data Availability

The raw data supporting the conclusions of this article will be made available by the authors, without undue reservation.
