# Supplementary material for: Association of shift work with metabolic dysfunction-associated fatty liver disease among subway workers
Source: Front Public Health. 2025 Dec 29;13:1737770. doi: 10.3389/fpubh.2025.1737770 (PMC12791039; doi:10.3389/fpubh.2025.1737770)
Supplement: Supplementary file 1 [file Supplementary_file_1.docx]

***Supplementary Material***

# **1 Supplementary Figures and Tables**

## **1.1 Supplementary Figures**

**Supplementary Figure**
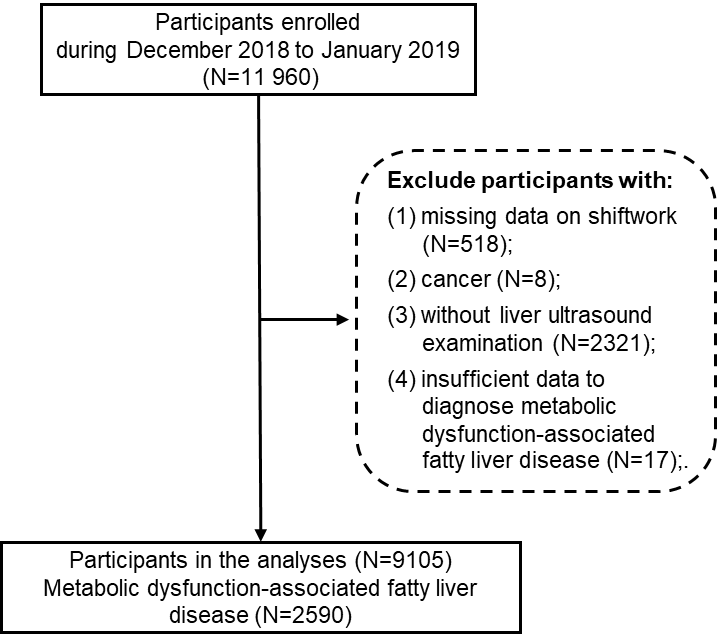
 **S1. Flowchart for the selection of participants**

**Supplementary**
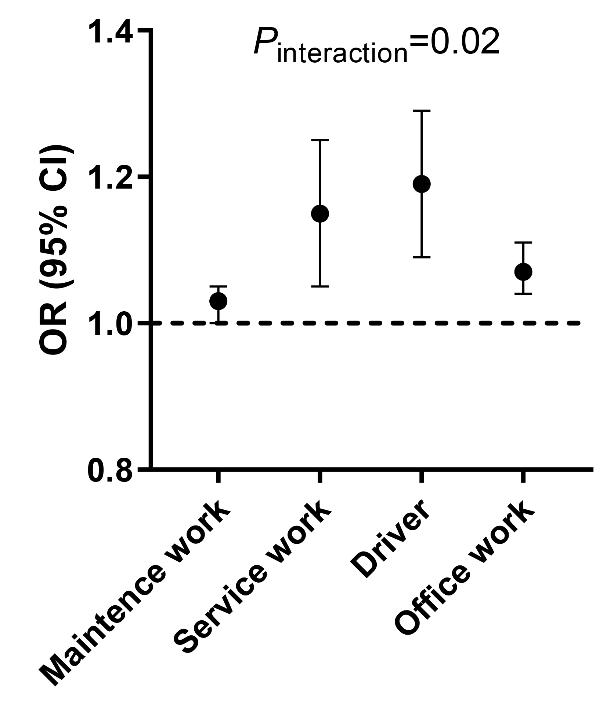
**Figure S2.** **Association between shift work duration and metabolic dysfunction-associated fatty liver disease stratified by job category.** Shift work duration was included in the models as a continuous variable; Models adjusted for age, sex, educational status, smoking status, drinking status, sleep duration, sleep quality, dietary consumption of grain, beans or soy products, vegetables and fruits, milk or dairy products, meat, fish or seafood, and egg, hypertension, dyslipidemia, and diabetes.

## **1.2 Supplementary Tables**

| **Table S1 Basic characteristics of the study population according to the status of shift work** | | | |
| --- | --- | --- | --- |
| **Variables** | **No shift work** | **Shift work** | ***P* value^a^** |
| N | 2699 | 6406 |  |
| Age, years | 28.1 ± 5.2 | 26.8 ± 3.7 | <0.001 |
| Female, n (%) | 233 (8.6) | 1587 (24.8) | <0.001 |
| Body mass index^b^, kg/m^2^ | 23.7 ± 3.6 | 23.2 ± 3.7 | <0.001 |
| Education level^b^, n (%) |  |  | <0.001 |
| High school or below | 11 (0.4) | 36 (0.6) |  |
| University/college | 2493 (92.4) | 6280 (98.0) |  |
| Graduate student or above | 168 (6.2) | 14 (0.2) |  |
| Smoking status^b^, n (%) |  |  | 0.04 |
| Never smoker | 1980 (73.4) | 4844 (75.6) |  |
| Current smoker | 610 (22.6) | 1362 (21.3) |  |
| Former smoker | 99 (3.7) | 185 (2.9) |  |
| Drinking status^b^, n (%) |  |  | <0.001 |
| Never drinker | 2042 (75.7) | 5202 (81.2) |  |
| Current drinker | 571 (21.2) | 1087 (17.0) |  |
| Former drinker | 78 (2.9) | 104 (1.6) |  |
| Active exercise^b^, n (%) | 446 (16.5) | 849 (13.3) | <0.001 |
| Sleep duration^b^, h | 7.3±1.1 | 7.7±1.6 | <0.001 |
| Sleep quality^b^, n (%) |  |  | <0.001 |
| Good | 784 (29.0) | 1390 (21.7) |  |
| Fair | 1477 (54.7) | 3580 (55.9) |  |
| Poor | 198 (7.3) | 879 (13.7) |  |
| Diet categories (≥5 times/week)^b^, n (%) | |  |  |
| Grain | 2628 (97.4) | 6253 (97.6) | 0.07 |
| Beans or soy products | 637 (23.6) | 1765 (27.6) | <0.001 |
| Vegetables and fruits | 2306 (85.4) | 5492 (85.7) | 0.23 |
| Milk or dairy products | 794 (29.4) | 2448 (38.2) | <0.001 |
| Meat | 2218 (82.2) | 5295 (82.7) | 0.53 |
| Fish or seafood | 381 (14.1) | 1208 (18.9) | <0.001 |
| Egg | 1114 (41.3) | 2934 (45.8) | <0.001 |
| Job category, n (%) |  |  | <0.001 |
| Maintence work | 2010 (74.5) | 2297 (35.9) |  |
| Service work | 18 (0.7) | 1281 (20.0) |  |
| Driver | 43 (1.6) | 1302 (20.3) |  |
| Office work | 628 (23.3) | 1526 (23.8) |  |
| Hypertension^b^, n (%) | 287 (10.6) | 606 (9.5) | 0.06 |
| Dyslipidemia^b^, n (%) | 587 (21.7) | 1075 (16.8) | <0.001 |
| Diabetes^b^, n (%) | 39 (1.4) | 52 (0.8) | 0.005 |
| Continuous variables are presented as mean ± SD. Categorical variables are presented as numbers (percentages). | | | |
| ^a^*P* values were estimated using student t test for continuous variables, and Chi-square tests for categorical variables. | | | |
| ^b^Data were incomplete for these variables. A total of 21 (0.2%), 103 (1.1%), 25 (0.3%), 21 (0.2%), 201 (2.2%), 141 (1.6%), 797 (8.8%), 125 (1.4%), 553 (6.1%), 336 (3.7%), 493 (5.4%), 373 (4.1%), 565 (6.2%), 474 (5.2%), 544 (6.0%), 501 (5.5%), and 608 (6.7 %) participants had missing value of BMI, education level, smoking status, drinking status, active exercise, sleep duration, sleep quality, diet frequency of grain, beans or soy products, vegetables and fruits, milk or dairy products, meat, fish or sea food, and egg, hypertension, dyslipidemia, and diabetes. | | | |

| **Table S2 Association between shift work and metabolic dysfunction-associated fatty liver disease after excluding participants with missing values** | | | |
| --- | --- | --- | --- |
| **Variables** | **Cases/total population** | **OR (95% CI)** | ***P* value** |
| Duration of shift work, years | |  |  |
| No shift work | 620/2006 | 1.00 (Ref) |  |
| ≤3 | 322/1870 | 0.77 (0.63, 0.93) | 0.006 |
| >3-6 | 524/1894 | 1.23 (1.04, 1.46) | 0.016 |
| >6 | 471/1142 | 1.49 (1.24, 1.79) | <0.001 |
| per 1 year increase | 1937/6912 | 1.05 (1.03, 1.08) | <0.001 |
| Shift work type |  |  |  |
| No shift work | 620/2006 | 1.00 (Ref) |  |
| Two-shift | 292/842 | 1.14 (0.94, 1.39) | 0.171 |
| Three-shift | 712/2855 | 1.15 (0.97, 1.37) | 0.113 |
| Four-shift | 313/1209 | 1.15 (0.95, 1.39) | 0.154 |
| Models adjusted for age, sex, education level, smoking status, drinking status, active exercise, sleep duration, sleep quality, dietary consumption of grain, beans or soy products, vegetables and fruits, milk or dairy products, meat, fish or seafood, and egg, job category, hypertension, dyslipidemia, and diabetes. | | | |

| **Table S3 Association of shift work duration with metabolic dysfunction-associated fatty liver disease among subgroups** | | | | | |
| --- | --- | --- | --- | --- | --- |
| **Variables** | **Shift work duration, years** | | | | ***P*_interaction_^a^** |
|  | **No shift work** | **0-3** | **>3-6** | **>6** |  |
| **Sex** |  |  |  |  | 0.53 |
| Male |  |  |  |  |  |
| Cases/total population | 824/2466 | 422/1846 | 619/1762 | 582/1211 |  |
| OR (95% CI) | 1.00 (Ref) | 0.78 (0.67, 0.92) | 1.22 (1.05, 1.41) | 1.58 (1.34, 1.87) |  |
| Female |  |  |  |  |  |
| Cases/total population | 23/233 | 28/635 | 42/645 | 50/307 |  |
| OR (95% CI) | 1.00 (Ref) | 1.05 (0.50, 2.19) | 1.30 (0.67, 2.52) | 1.96 (1.04, 3.69) |  |
| **Body mass index, kg/m^2^** |  |  |  |  | 0.59 |
| <24 |  |  |  |  |  |
| Cases/total population | 95/1540 | 52/1760 | 78/1509 | 68/762 |  |
| OR (95% CI) | 1.00 (Ref) | 0.85 (0.58, 1.26) | 1.27 (0.90, 1.79) | 1.52 (1.06, 2.19) |  |
| ≥24 |  |  |  |  |  |
| Cases/total population | 752/1155 | 398/717 | 583/891 | 563/750 |  |
| OR (95% CI) | 1.00 (Ref) | 0.81 (0.65, 1.01) | 1.22 (0.99, 1.51) | 1.64 (1.31, 2.07) |  |
| **Current smoker** |  |  |  |  | 0.39 |
| No |  |  |  |  |  |
| Cases/total population | 584/2079 | 330/2042 | 482/1929 | 394/1058 |  |
| OR (95% CI) | 1.00 (Ref) | 0.81 (0.67, 0.97) | 1.28 (1.08, 1.51) | 1.65 (1.37, 2.00) |  |
| Yes |  |  |  |  |  |
| Cases/total population | 260/610 | 120/430 | 179/475 | 236/457 |  |
| OR (95% CI) | 1.00 (Ref) | 0.77 (0.55, 1.07) | 1.01 (0.75, 1.35) | 1.44 (1.08, 1.92) |  |
| **Current drinker** |  |  |  |  | 0.67 |
| No |  |  |  |  |  |
| Cases/total population | 647/2120 | 381/2119 | 522/2005 | 475/1182 |  |
| OR (95% CI) | 1.00 (Ref) | 0.85 (0.71, 1.01) | 1.21 (1.03, 1.43) | 1.65 (1.38, 1.98) |  |
| Yes |  |  |  |  |  |
| Cases/total population | 197/571 | 68/358 | 139/398 | 156/331 |  |
| OR (95% CI) | 1.00 (Ref) | 0.57 (0.39, 0.83) | 1.23 (0.89, 1.69) | 1.46 (1.05, 2.02) |  |
| **Active exercise** |  |  |  |  | 0.84 |
| No |  |  |  |  |  |
| Cases/total population | 691/2196 | 380/2062 | 549/2038 | 549/1313 |  |
| OR (95% CI) | 1.00 (Ref) | 0.85 (0.72, 1.02) | 1.22 (1.04, 1.43) | 1.62 (1.36, 1.93) |  |
| Yes |  |  |  |  |  |
| Cases/total population | 143/446 | 62/359 | 98/316 | 76/174 |  |
| OR (95% CI) | 1.00 (Ref) | 0.55 (0.37, 0.83) | 1.19 (0.82, 1.71) | 1.51 (1.00, 2.29) |  |
| **Sleep duration, h** |  |  |  |  | 0.03 |
| <7 |  |  |  |  |  |
| Cases/total population | 468/1353 | 213/938 | 324/926 | 316/742 |  |
| OR (95% CI) | 1.00 (Ref) | 0.73 (0.59, 0.92) | 1.22 (1.00, 1.50) | 1.45 (1.17, 1.80) |  |
| ≥7 |  |  |  |  |  |
| Cases/total population | 359/1297 | 226/1510 | 326/1449 | 300/749 |  |
| OR (95% CI) | 1.00 (Ref) | 0.85 (0.67, 1.07) | 1.18 (0.96, 1.46) | 1.68 (1.32, 2.13) |  |
| **Hypertension** |  |  |  |  | 0.39 |
| No |  |  |  |  |  |
| Cases/total population | 614/2234 | 328/2112 | 525/2071 | 482/1251 |  |
| OR (95% CI) | 1.00 (Ref) | 0.80 (0.67, 0.96) | 1.28 (1.09, 1.50) | 1.67 (1.41, 1.99) |  |
| Yes |  |  |  |  |  |
| Cases/total population | 171/287 | 89/225 | 108/213 | 112/168 |  |
| OR (95% CI) | 1.00 (Ref) | 0.71 (0.46, 1.10) | 0.96 (0.62, 1.46) | 1.39 (0.84, 2.28) |  |
| **Dyslipidemia** |  |  |  |  | 0.84 |
| No |  |  |  |  |  |
| Cases/total population | 430/1952 | 253/2034 | 385/1891 | 325/1065 |  |
| OR (95% CI) | 1.00 (Ref) | 0.77 (0.64, 0.94) | 1.24 (1.04, 1.47) | 1.52 (1.26, 1.84) |  |
| Yes |  |  |  |  |  |
| Cases/total population | 373/587 | 171/310 | 254/399 | 278/366 |  |
| OR (95% CI) | 1.00 (Ref) | 0.87 (0.62, 1.21) | 1.20 (0.89, 1.61) | 1.74 (1.26, 2.40) |  |
| Models adjusted for age, sex, education level, smoking status, drinking status, active exercise, sleep duration, sleep quality, dietary consumption of grain, beans or soy products, vegetables and fruits, milk or dairy products, meat, fish or seafood, and egg, job category, hypertension, dyslipidemia, and diabetes, except for the corresponding stratified factor. | | | | | |
| ^a^The test for interaction was between the continuous duration of shift work and the stratified variable. | | | | | |

| **Table S4 Association of shift work types with metabolic dysfunction-associated fatty liver disease among subgroups** | | | | | |
| --- | --- | --- | --- | --- | --- |
| **Variables** | **Shift work type** | | | | ***P*_interaction_** |
|  | **No shift work** | **Two-shift** | **Three-shift** | **Four-shift** |  |
| **Sex** |  |  |  |  | 0.95 |
| Male |  |  |  |  |  |
| Cases/total population | 824/2466 | 359/1031 | 865/2610 | 399/1178 |  |
| OR (95% CI) | 1.00 (Ref) | 1.11 (0.94, 1.32) | 1.16 (1.01, 1.34) | 1.18 (1.00, 1.40) |  |
| Female |  |  |  |  |  |
| Cases/total population | 23/233 | 8/64 | 73/1075 | 39/448 |  |
| OR (95% CI) | 1.00 (Ref) | 1.86 (0.70, 4.91) | 1.47 (0.79, 2.75) | 1.62 (0.82, 3.18) |  |
| **Body mass index, kg/m^2^** |  |  |  |  | 0.89 |
| <24 |  |  |  |  |  |
| Cases/total population | 95/1540 | 40/622 | 107/2380 | 51/1029 |  |
| OR (95% CI) | 1.00 (Ref) | 1.09 (0.73, 1.64) | 1.22 (0.88, 1.70) | 1.30 (0.88, 1.92) |  |
| ≥24 |  |  |  |  |  |
| Cases/total population | 752/1155 | 327/473 | 830/1296 | 387/589 |  |
| OR (95% CI) | 1.00 (Ref) | 1.22 (0.95, 1.56) | 1.16 (0.95, 1.41) | 1.22 (0.96, 1.54) |  |
| **Current smoker** |  |  |  |  | 0.90 |
| No |  |  |  |  |  |
| Cases/total population | 584/2079 | 254/813 | 646/2927 | 306/1289 |  |
| OR (95% CI) | 1.00 (Ref) | 1.16 (0.95, 1.42) | 1.20 (1.02, 1.40) | 1.19 (0.98, 1.43) |  |
| Yes |  |  |  |  |  |
| Cases/total population | 260/610 | 113/280 | 291/749 | 131/333 |  |
| OR (95% CI) | 1.00 (Ref) | 1.05 (0.75, 1.46) | 1.08 (0.83, 1.42) | 1.20 (0.87, 1.66) |  |
| **Current drinker** |  |  |  |  |  |
| No |  |  |  |  |  |
| Cases/total population | 647/2120 | 283/863 | 734/3064 | 361/1379 |  |
| OR (95% CI) | 1.00 (Ref) | 1.15 (0.95, 1.39) | 1.18 (1.01, 1.38) | 1.25 (1.04, 1.49) |  |
| Yes |  |  |  |  |  |
| Cases/total population | 197/571 | 84/230 | 203/613 | 76/244 |  |
| OR (95% CI) | 1.00 (Ref) | 1.03 (0.71, 1.49) | 1.16 (0.86, 1.56) | 1.01 (0.69, 1.47) |  |
| **Active exercise** |  |  |  |  |  |
| No |  |  |  |  | 0.59 |
| Cases/total population | 691/2196 | 311/907 | 793/3127 | 374/1379 |  |
| OR (95% CI) | 1.00 (Ref) | 1.17 (0.97, 1.41) | 1.19 (1.03, 1.39) | 1.25 (1.05, 1.50) |  |
| Yes |  |  |  |  |  |
| Cases/total population | 143/446 | 53/174 | 128/471 | 55/204 |  |
| OR (95% CI) | 1.00 (Ref) | 0.97 (0.63, 1.49) | 1.02 (0.72, 1.44) | 0.98 (0.64, 1.50) |  |
| **Sleep duration, h** |  |  |  |  | 0.61 |
| <7 |  |  |  |  |  |
| Cases/total population | 468/1353 | 184/528 | 451/1406 | 218/672 |  |
| OR (95% CI) | 1.00 (Ref) | 1.01 (0.80, 1.29) | 1.14 (0.95, 1.38) | 1.17 (0.94, 1.47) |  |
| ≥7 |  |  |  |  |  |
| Cases/total population | 359/1297 | 175/547 | 471/2241 | 206/920 |  |
| OR (95% CI) | 1.00 (Ref) | 1.22 (0.95, 1.57) | 1.16 (0.95, 1.42) | 1.16 (0.91, 1.49) |  |
| **Hypertension** |  |  |  |  | 0.31 |
| No |  |  |  |  |  |
| Cases/total population | 614/2234 | 283/898 | 728/3193 | 324/1343 |  |
| OR (95% CI) | 1.00 (Ref) | 1.22 (1.01, 1.47) | 1.20 (1.03, 1.40) | 1.25 (1.04, 1.50) |  |
| Yes |  |  |  |  |  |
| Cases/total population | 171/287 | 68/138 | 158/302 | 83/166 |  |
| OR (95% CI) | 1.00 (Ref) | 0.78 (0.48, 1.25) | 1.04 (0.69, 1.58) | 1.03 (0.65, 1.65) |  |
| **Dyslipidemia** |  |  |  |  | 0.23 |
| No |  |  |  |  |  |
| Cases/total population | 430/1952 | 197/799 | 531/2925 | 235/1266 |  |
| OR (95% CI) | 1.00 (Ref) | 1.16 (0.94, 1.42) | 1.15 (0.98, 1.35) | 1.10 (0.90, 1.33) |  |
| Yes |  |  |  |  |  |
| Cases/total population | 373/587 | 155/235 | 377/593 | 171/247 |  |
| OR (95% CI) | 1.00 (Ref) | 1.14 (0.81, 1.60) | 1.24 (0.94, 1.64) | 1.46 (1.03, 2.08) |  |
| Models adjusted for age, sex, education level, smoking status, drinking status, active exercise, sleep duration, sleep quality, dietary consumption of grain, beans or soy products, vegetables and fruits, milk or dairy products, meat, fish or seafood, and egg, job category, hypertension, dyslipidemia, and diabetes, except for the corresponding stratified factor. | | | | | |
